# Insights into Successful Hydrothermal Synthesis of Brookite TiO_2_ Particles: From Micro to Nano

**DOI:** 10.1021/acsomega.5c06112

**Published:** 2025-11-04

**Authors:** Luke T. Coward, Nataliya Stynka, Victoria G. Magyar, Ava Foreman, Luca Antonescu, Hanna McFadden, Lorelei Dippy, Jocelyn D. Shutak, Madeline Kesner, Shawn Overcash, Joshua Davis, Camila Rendon Bernot, Oksana Love

**Affiliations:** 8620 University of North Carolina Asheville; One University Heights, Asheville North Carolina 28804, United States

## Abstract

In this study, we investigate the hydrothermal synthesis of brookite phase TiO_2_, resulting in the successful formation of flower-like microstructures and rod-like nanoparticles. In this method, a pH of 12.5 is critical for initiating the formation of the brookite phase prior to crystallization. Specifically, Na^+^ cations facilitate interactions between titanate layers, which promote this transition. Our findings indicate that a threshold concentration of Na^+^ ions is required to stabilize the brookite structure; deviations below this concentration instead favor the formation of anatase. Notably, we demonstrate that an optimal Na^+^ level enables the reliable formation of rod-like, ligand-free brookite nanoparticles. Photodegradation tests conducted with trichloroethylene as a representative pollutant showed promising photocatalytic efficiency for both structural forms, with the rod-like nanoparticles exhibiting superior activity.

## Graphical abstract

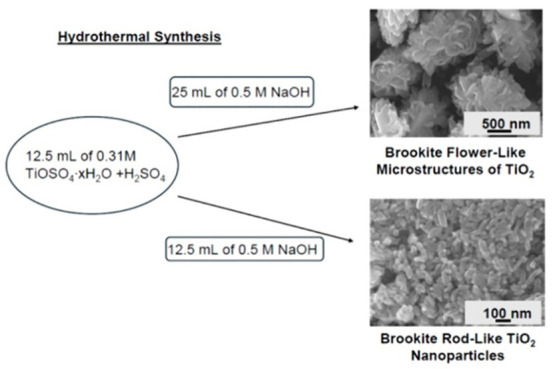

## 1 Introduction

Titanium dioxide, TiO_2_, has been widely tested in photocatalytic applications such as solar cells, pollutant degradation, or valuable chemical production.^1–3^ These processes generate and transport free charge carriers (electrons and holes) created by UV–visible light absorption. The low-wavelength photon energies correspond to the wide band gap of crystalline TiO_2_ nanoparticles with overlapping molecular orbitals.^4–6^ TiO_2_ exists in three primary crystal phases: anatase, rutile, and brookite (Figure 1). Anatase and rutile both adopt tetragonal crystal structures; anatase consists of corner-sharing TiO_6_ octahedra forming (001) planes, while rutile features edge-sharing octahedra along the same (001) planes.^7,8^ In contrast, brookite has an orthorhombic structure composed of both edge- and corner-sharing octahedra.^9^ Each polymorph exhibits a distinct band gap: brookite (∼3.4 ± 0.1 eV) has the widest, followed by anatase (∼3.2 ± 0.1 eV)^10^ and rutile (∼3.0 ± 0.1 eV).^9,10^ Most research has focused on anatase and rutile, particularly Degussa P25 (a commercially available mixture of 75% anatase and 25% rutile) that has been extensively investigated over the past decades.^7^ Brookite remains the least explored photocatalyst due to its past synthetic challenges.

**Figure 1:**
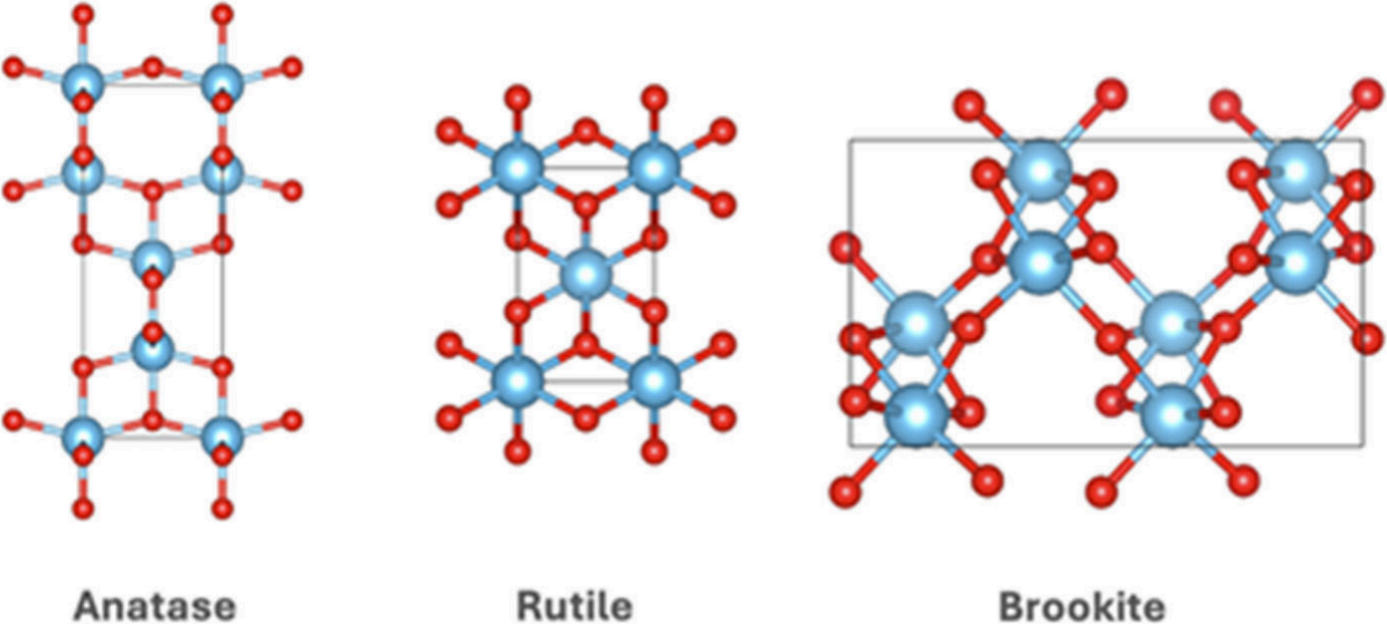
Unit cells of TiO_2_: anatase, rutile, and brookite.

The brookite phase can be synthesized by using a variety of approaches. They include the hydrothermal method, solvothermal method, sol–gel method, precipitation method, high temperature amine hydrolysis method, and many others.^11^ However, the two most commonly used methods for producing the brookite structure are solvothermal and hydrothermal. The final brookite structure can be tuned by varying different parameters within the methods, such as type of solvent, pH, type of precursor, type of ligands, etc.^12^ Hydrothermal synthesis is favored for TiO_2_ preparation because of its numerous advantageous features, such as good crystallinity, high product purity, and crystal symmetry.^13^ In this method, an autoclave is used to seal typically an aqueous solution of titanium source, and it is placed in an oven or furnace at a specific temperature and time. According to a recent review, various titanium precursors, such as TiO_2_ nanoparticles, TiCl_4_, titanium foil, and titanium hydroxide particles can be used to form brookite structures.^11^ In addition to the choice of precursors, numerous studies have investigated the synthesis of pure brookite phase TiO_2_ by incorporating various ligands during hydrothermal processing. For example, Truong and co-workers were able to synthesize brookite nanoparticles by using amino acids as the structure-directing and shape-controlling agents.^14^ Katsumata and co-workers used glycolic acid as their complexant.^15^ And Yu and co-workers utilized PVP to prepare flower-like brookite TiO_2_ nanostructures,^16^ while Li and co-workers created high quality brookite single-crystalline nanosheets with sodium lactate as the complexant and surfactant.^17^ Nevertheless, many research groups have intentionally avoided using ligands or surfactants in order to synthesize the brookite phase, specifically in a hydrothermal approach. For example, Hu and co-workers showed that NaOH is an excellent pH modifier when paired with a water-soluble titanyl sulfate precursor (TiOSO_4_), leading to brookite TiO_2_ microstructures where irregular faceting causes the size and shape of nanomaterials to create flower-like structures, if given enough time for Ostwald ripening to occur during autoclave treatment.^18^ Zou and co-workers expanded on this work, showing that TiCl_4_ and NaOH based precipitation reactions autoclaved under different temperatures (200 °C, 240 °C) will select for different brookite microstructure morphologies: flower- or rice-like.^16^

In general, three distinct types of hydrothermal syntheses can be identified: first, synthesis under strongly acidic conditions, second, synthesis using layered titanates under basic conditions, and last, synthesis using water-dissolved Ti species under basic pH.^19^ This study explores the second type of synthesis within the hydrothermal method, where the precursor TiOSO_4_ is used as a titanium source and NaOH as a source of OH^–^ and Na^+^ ions. Interestingly, brookite phase formation not only requires basic conditions but also critically depends on the presence of Na^+^ ions.^20^ Even in the presence of aqueous ammonia as the base, Na^+^ ions have been found to stabilize the layered titanate structure that leads to formation of brookite form.^21^

Flower-like brookite structures have been synthesized and investigated by numerous research groups.^16,18,22^ Studies have shown that this type of morphology exhibits superior photocatalytic activity compared to brookite formed by aggregated nanoparticles in micrometer-sized clusters.^16^ Through the systematic investigation of pH adjustment via NaOH and varying heating durations, this study supports the key role of Na^+^ ions in the aqueous hydrothermal synthesis of brookite TiO_2_. We demonstrate how these parameters affect the structure and size of the resulting brookite nanoparticles. Notably, we report the successful synthesis of rod-like brookite nanoparticles without the use of protective ligands, which exhibit promising performance in rapid trichloroethylene (TCE) photodegradation tests.

## 2 Results and Discussion

Before delving into the pH effect, heating temperature analysis, or NaOH concentration effect, it is essential to examine our general synthetic hydrothermal method for the brookite phase of TiO_2_ particles. First the Ti precursor TiOSO_4_·*x*H_2_O + H_2_SO_4_ is dissolved in water. In this step, an acidic transparent solution of TiO_2_ is created (Figure 2, Step 1). The subsequent addition of a specific concentration of aqueous NaOH raises the pH of the solution (Figure 2, Step 2) and initiates a precipitation reaction, resulting in the formation of visible white TiO_2_ sol/amorphous titanates. Higher pH level and increased NaOH concentrations promote sol formation, ultimately producing a greater yield of particles. After the solution is stirred overnight, it is purified via centrifugation (Figure 2, Step 3), and the pH must be adjusted a second time to promote brookite phase formation (Figure 2, Step 4). Subsequently, the sample is placed into an autoclave for a specific duration at 220 °C (Figure 2, Step 5); afterward, particles are filtered and ready to be analyzed (Figure 2, Step 6).

**Figure 2:**
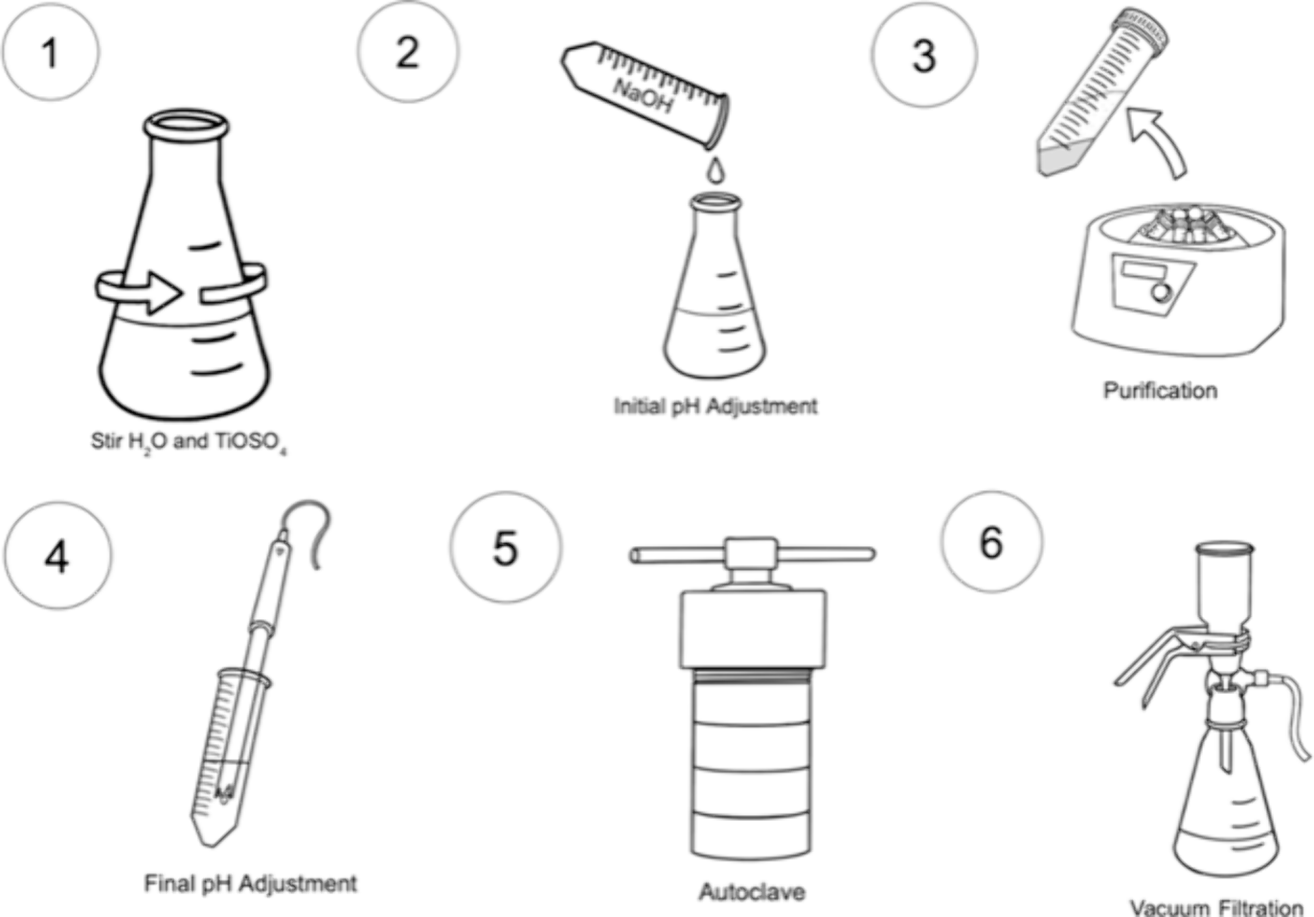
Step-by-step procedure of synthesizing brookite TiO_2_ nanoparticles.

### 2.1 Final pH Adjustments for Brookite TiO_2_ Formation

To study how the final pH adjustment influences brookite TiO_2_ formation, the final pH values (Figure 2, Step 4) were adjusted to 1, 6, 7, 10.5, and 12.5. Only at pH ∼12.5 was pure brookite synthesized. The characteristic peaks of brookite include the (210), (111) doublet peak at 2θ = 25.4° and 25.7° as well as the singlet peak at 2θ = 30.8° corresponding to the (211) plane (Figure 3A), which are all in good agreement with that of purchased brookite nanoparticles, and COD reference patterns (Figure S1). The content of the brookite structure for the pH 12.5 sample was 100% as calculated by XRD data refinement. At pH 10.5, the diffraction pattern showed that a mixture of brookite and anatase had formed (Figure S2), whereas samples at pH 6 and pH 7 formed pure anatase phase TiO_2_ (Figure 3B and 3C). The characteristic (101) peak for anatase present at 2θ = 25.3° and the triple peaks corresponding to the (103), (004), (112) planes around 2θ = 37.8° aligned well with purchased anatase and the COD reference pattern (Figure S1). The sample at pH 1, on the other hand, consistently produced a mixture of anatase and rutile structures (Figure 3D), more closely resembling the XRD pattern of P25 particles (Figure S1). It is important to note that our results align well with literature values, but we did not observe the formation of titanate sheets in our final products. This may be attributed to the final pH adjustment using HNO_3_, as the solution is generally basic, even after purification/centrifugation. It is also noteworthy that only NaOH has been effective in producing the brookite structure, consistent with previous literature^16^ reporting that KOH results exclusively in anatase formation (Figure S3). Literature reports suggest that layered titanate structures form during the initial stages of synthesis, with Na^+^ ions stabilizing these layers and facilitating their transformation into brookite phase.^16,17^ Our observations strongly suggest that to hydrothermally obtain the brookite phase TiO_2_ for our suggested synthetic method; final pH prior to crystallization must be adjusted to ∼12.5 with NaOH.

**Figure 3:**
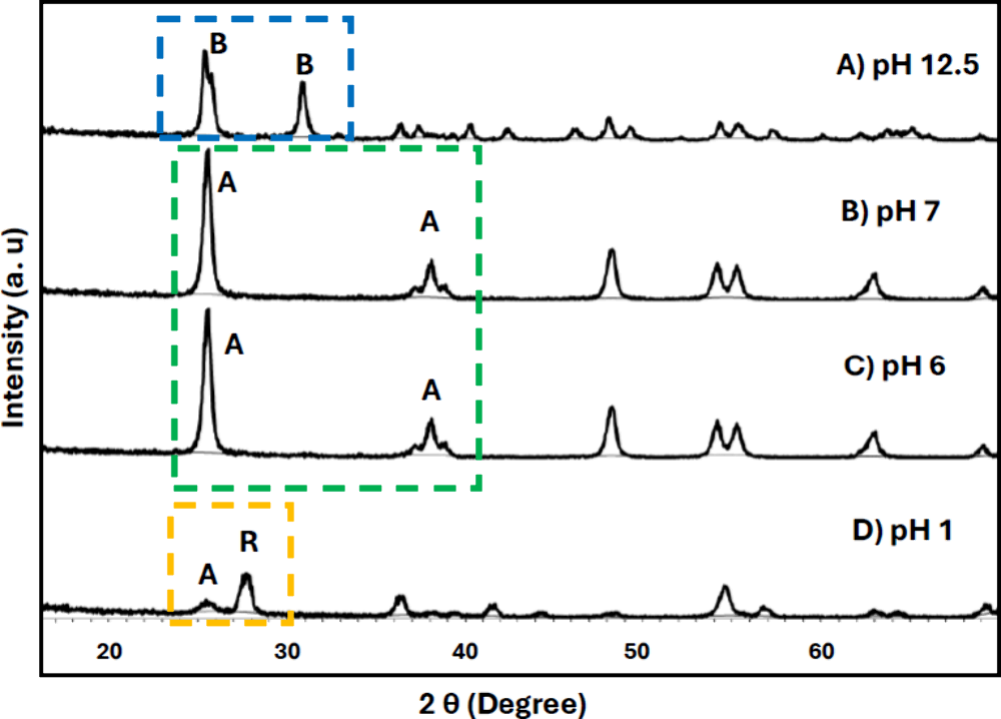
XRD patterns of the samples prepared at 220 °C for 24 h heating time with final pH adjustment to (A) pH 12.5 - brookite (B), (B) pH 7 - anatase (A), (C) pH 6 - anatase (A), and (D) pH 1 - mixture of anatase (A) and rutile (R).

### 2.2 Heating Time Effects on Crystal Structure

To assess the effects of heating time on the crystal phase of TiO_2_ particles, all samples were heated at 220 °C for 24, 48, 72, and 96 h. For pH 1 samples, an increase in anatase character was observed with longer heating times (Figure S4), while no phase changes were observed for pH 6 and pH 7 samples at all heating durations (Figure S5). It is noteworthy that our attempts to heat the samples for shorter than 24 h durations resulted in amorphous structures, with no crystalline phases observed. At 24 h of heating using 150 °C, the synthesis yielded amorphous products, whereas extended heating times beyond 24 h at those temperatures led to the formation of the anatase phase (not pictured here). Temperature increases beyond 220 °C were restricted by the thermal stability of the autoclave inserts; thus, all samples were heated at 220 °C.

Given that the pH studies confirmed formation of a pure phase brookite structure only when the final pH was adjusted to ∼12.5, our discussions herein will remain centered on those brookite samples. Figure 4 displays the XRD patterns of samples prepared at pH 12.5 that were heated at 220 °C for 24, 48, 72, and 96 h. At all heating durations, the samples exhibited pure brookite phase with high crystallinity (<85%). All samples exhibited flower-like morphologies composed of aggregated fine particles forming microstructures, with no notable variation in surface texture (Figure S6); however, an increase in heating time correlated with the growth of bigger structural features creating even larger flower-like microstructures at 96 h of heating (Figure 5).

**Figure 4:**
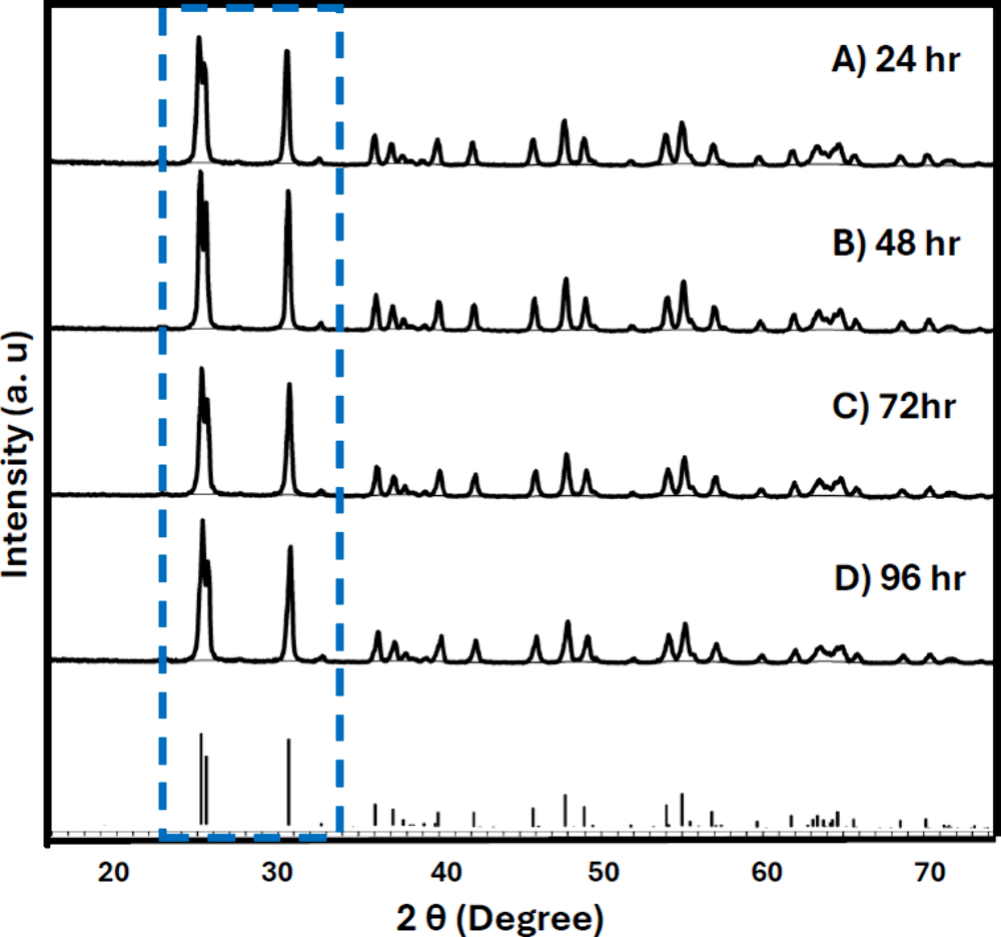
XRD patterns of the samples prepared at final pH 12.5 heated at (A) 24 h, (B) 48 h, (C) 72 h, and (D) 96 h. COD 9004140 for brookite.

**Figure 5:**
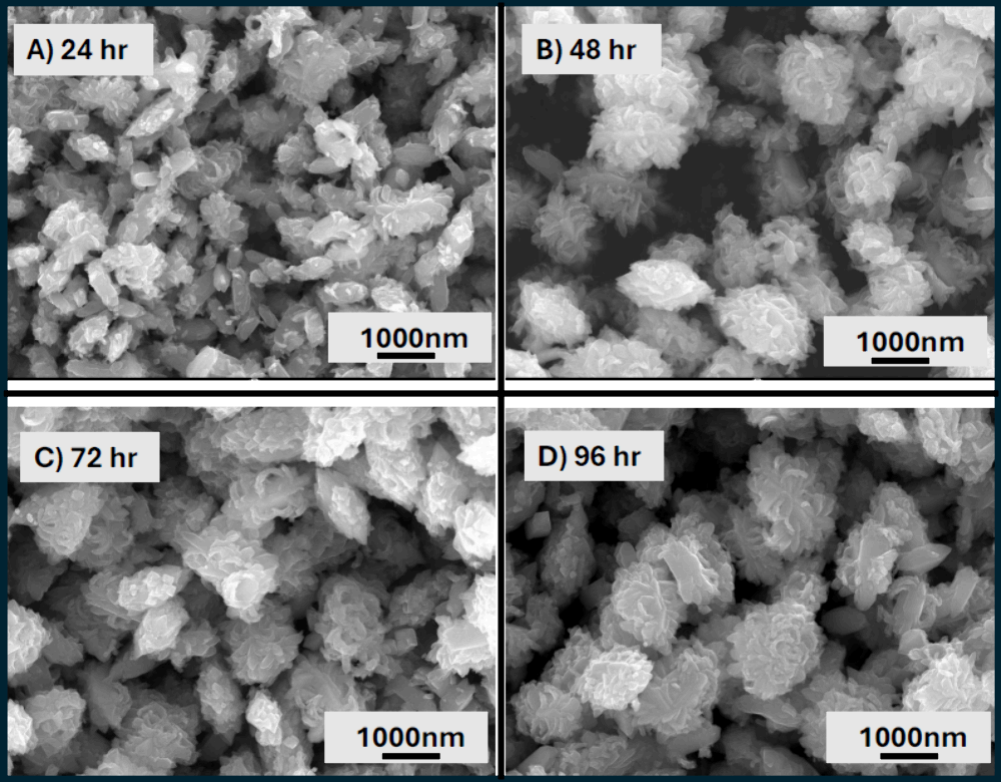
SEM images of brookite TiO_2_ particles at (A) 24 h, (B) 48 h, (C) 72 h, and (D) 96 h of heating at 220 °C.

### 2.3 Effect of NaOH (aq) Concentration on the Size and Phase of TiO_2_ Particles

As previously noted, in the synthetic procedure (Figure 2, Step 2) NaOH is added to the transparent acidic solution of TiO_2_ to produce sol/titanates. As stated in the experimental section, optimal sol production is achieved by adding 25 mL of 0.5 M NaOH, which adjusts the pH to approximately 12.5. After synthesis is complete, the final particles create flower-like agglomerates in the microscale range (Figure 5). To assess the importance of this step in the procedure, we examined how varying concentrations of Na^+^ ions influence particle formation and the corresponding crystal structures. Different volumes of 0.5 M NaOH solution (e.g., 6, 12.5, 25, and 50 mL), as well as 12.5 mL of 1 M NaOH, were added to 12.5 mL of 0.31 M TiOSO_4_·*x*H_2_O + H_2_SO_4_ to achieve final NaOH concentrations of 0.16, 0.25, 0.33, 0.4, and 0.5 M, respectively. In these experiments, the addition of varying volumes of NaOH resulted in different pH values for sol/titanates formation: pH ∼12.5 for the 0.33, 0.40, and 0.50 M solutions, around pH ∼7 for the 0.25 M sample, and pH ∼4 for 0.16 M solution. These pH differences led to slight variations in the amount of sol/titanates produced. The 0.16 M solution yielded the lowest mass, and the final nanoparticles (not shown here) produced a mixture of anatase and brookite phases (Figure 6A). The 0.25 M solution resulted in the formation of the brookite crystal phase (Figure 6B), exhibiting rod-like nanoparticles with an average diameter of 30 nm and lengths ranging from 40 to 140 nm (Figure 8). This has been the smallest brookite nanoparticle that we were able to synthesize without any protective ligands. The 0.33, 0.40, and 0.50 M solutions formed the brookite crystal structure and large flower-like particles. An increase in NaOH concentration led to larger agglomerate sizes ranging from 800 nm to 1.5 μm (Figure 7 and Figure S7). Although a noticeable increase in the overall particle size was observed, there was no significant variation in the average crystallite sizes across all samples (Table S1).

**Figure 6:**
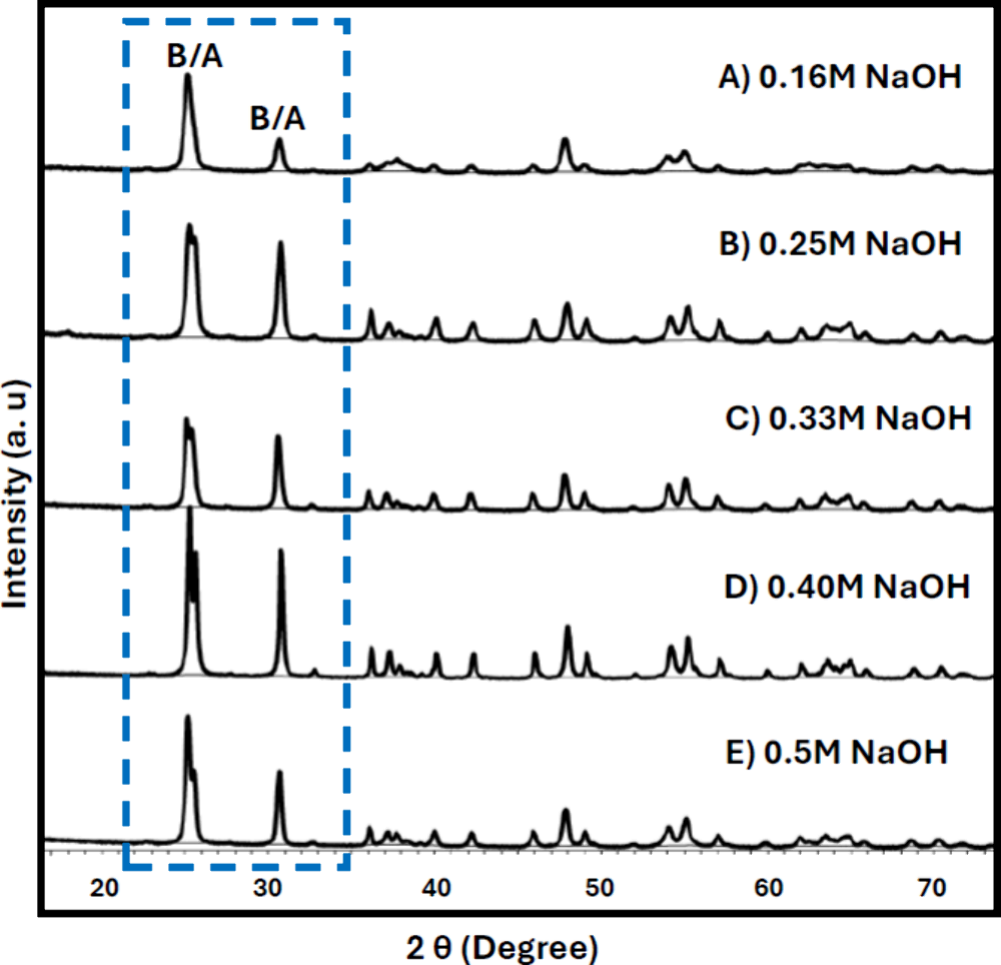
XRD patterns of the samples prepared with (A) 0.16 M NaOH, (B) 0.25 M NaOH, (C) 0.33 M NaOH, (D) 0.40 M NaOH, and (E) 0.50 M NaOH.

**Figure 7:**
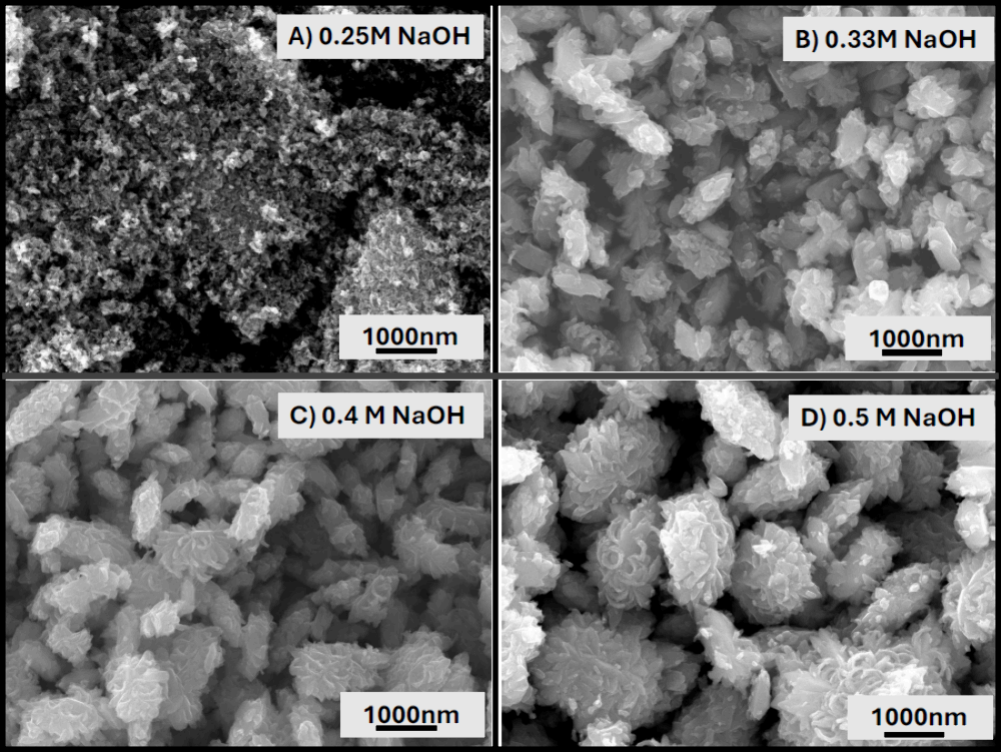
SEM images of the samples prepared with 12.5 mL of 0.31 M TiOSO_4_·*x*H_2_O + H_2_SO_4_ and (A) final 0.25 M NaOH obtained from addition of 0.5 M 12.5 mL NaOH, (B) final 0.33 M NaOH obtained from addition of 0.5 M 25 mL NaOH, (C) final 0.4 M NaOH obtained from addition of 0.5 M 50 mL NaOH, and (D) final 0.5 M NaOH obtained from addition of 1 M 12.5 mL NaOH. All samples were heated for 24 h at 220 °C and previously stated purification techniques were followed.

As mentioned previously, the literature suggests that Na^+^ ions stabilize layered titanate structures that facilitate sol transformation into the brookite phase when heating. Our data indicate that a threshold concentration of Na^+^ ions is necessary to form the brookite structure, and it is independent of the total volume of the solution whose variation generally only affects the size of particles. When the concentration of Na^+^ ions in the sol/titanates is insufficient, the separation of titanate layers is incomplete, resulting in a mixture of phases or the preferential formation of anatase. Our experimental results in previous sections clearly show that inadequate Na^+^ levels consistently favor the formation of anatase. This observation also helps clarify why the use of KOH instead of NaOH results in anatase formation. The larger size of K^+^ ions causes excessive spacing between titanate layers, preventing the structural arrangement required for brookite to form.

### 2.4 Photodegradation Test

The photocatalytic characteristics of the synthesized flower-like microstructures and rod-like brookite nanoparticles of TiO_2_ were examined by measuring the photodegradation of trichloroethylene (TCE), an industrial solvent that has been identified as a human carcinogen and contaminant by the US EPA.^23^ Photodegradation of TCE using TiO_2_ nanoparticles has been extensively studied over the past few decades, with most investigations focusing on the anatase phase or the commercially available P25 form of TiO_2_. To the best of our knowledge, no studies have reported the use of brookite TiO_2_ for TCE photodegradation. Previous studies using anatase-phase TiO_2_ have identified the formation of various intermediates, along with complete mineralization of TCE into CO_2_ and Cl^–^.^24^ The most commonly reported intermediates in the literature included molecules such HCl, Cl_2_, COCl_2_, ClCOCOCl, CHCl_3_, CHCl_2_COCl, and CHCl_2_CH_2_Cl.^25–27^

Prior to testing synthesized samples, purchased anatase and brookite nanoparticles (Figure S8) were used for TCE degradation. Degradation of TCE was calculated by measuring the concentration of TCE present before and after photocatalysis by GC/MS. Purchased brookite nanoparticles slightly outperformed anatase nanoparticles (Figure S9), and the reaction kinetics followed previously reported values in the literature.^28,29^ Therefore, in subsequent experiments, the synthesized flower-like particles (Figure 5A) and rod-like nanoparticles (Figure 8) were compared to commercially available brookite phase TiO_2_ nanoparticles (agglomerates). In these experiments, hardly any degradation of TCE was observed (<10%) when only UV light was applied with no TiO_2_ particles (Figure 9A) and no decrease in TCE concentration was detected when TiO_2_ particles were added to TCE solution but not illuminated (not shown here). Synthesized micro- and nanobrookite TiO_2_ particles outperformed purchased brookite nanoparticles. In 3 min, purchased brookite nanoparticles degraded approximately 70% of TCE (Figure 9B), while synthesized flower-like microstructures degraded almost 85% of TCE (Figure 9C). The rod-like nanoparticles completely degraded TCE in less than 1 min, with 50% degradation observed in 30 s. Although our setup could not quantify CO_2_ production, we observed no formation of TCE intermediates. Kinetic experiments were not conducted in this study, as they fall outside the project’s scope. However, they represent a promising direction for future investigations. The experiments performed served as a proof of concept, in which the nanoparticles exhibited faster degradation than flower-like particles (Figure 9D). These results may be attributed to factors previously reported in studies on anatase TiO_2_, notably the reduced particle size and increased surface area, both of which have been correlated with improved photocatalytic performance.^30^ Studies have demonstrated that Degussa P25 exhibits lower photocatalytic activity for TCE degradation compared with synthesized anatase nanoparticles, a trend consistent with our observations for both commercial and synthesized brookite samples. The photocatalytic efficiency of TiO_2_ was found to be highly dependent on particle size, as a decrease in size can induce notable changes in structural and electronic properties, thereby enhancing its catalytic performance.^31^ Similar photocatalytic behavior is observed in our study with the brookite phase, where smaller particle sizes demonstrate enhanced performance, likely attributed to a higher surface area-to-volume ratio and more efficient charge carrier dynamics.

**Figure 8:**
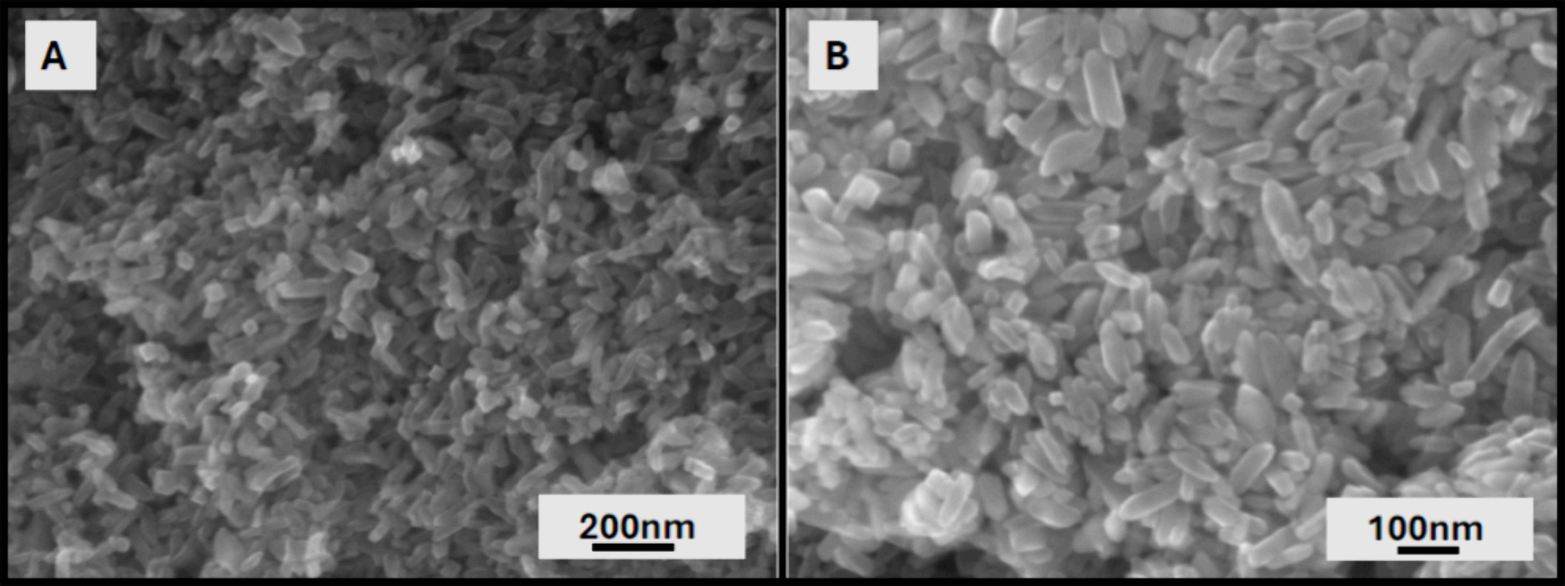
SEM images of 0.25 M NaOH sample with scale bars of (A) 200 and (B) 100 nm, respectively.

**Figure 9:**
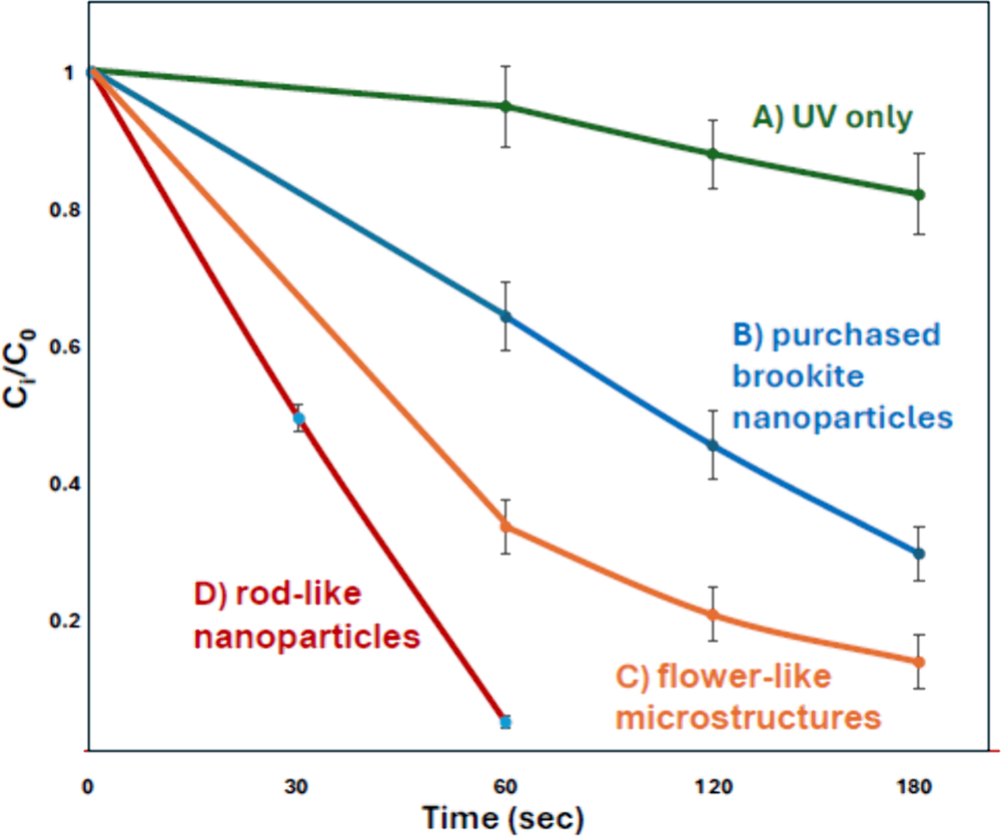
Photodegradation performance of TCE by (A) UV light only, (B) purchased brookite TiO_2_ nanoparticles, (C) synthesized brookite flower-like microstructures of TiO_2_, and (D) synthesized brookite rod-like TiO_2_ nanoparticles. Each data point was measured in triplicate, and the results are presented with the corresponding standard deviation.

## 3 Conclusions

In this study, hydrothermal TiO_2_ synthesis was utilized to produce flower-like microstructures, with a final pH adjustment using NaOH to around 12.5 identified as critical for the formation of the brookite phase. Heating time increased the size of flower-like structures but did not have an influence on the crystalline form. We further identified the optimal NaOH concentration for synthesizing ligand-free brookite TiO_2_ rod-like nanoparticles. Our results suggest that a specific Na^+^ threshold concentration is crucial for controlling nanoparticle size, as higher deviations from it tend to produce flower-like brookite structures, while lower concentration produces the anatase form. Photodegradation tests using TCE as a model pollutant demonstrated promising photocatalytic performance for both flower-like microstructures and rod-like nanoparticles, with particularly strong results for the rod-like morphology. To the best of our knowledge, this is the first report of ligand-free brookite rod-like nanoparticles synthesized via an aqueous hydrothermal method. This study demonstrates that subtle changes in synthetic conditions, specifically adjustments in Na^+^ concentration and the resulting pH, can significantly influence the final size of brookite TiO_2_ nanoparticles. These findings emphasize the importance of precise synthetic control in tuning the material properties for photocatalytic applications. Moreover, they lay the groundwork for the rational development of brookite-based photocatalysts with improved activity for a broader range of photocatalytic processes.

## 4 Experimental Section

### Materials

Anatase (15 nm), rutile (30 nm), and brookite crystals (1–100 nm) were purchased from US Research Nanomaterials, Inc. Degussa 25, titanium­(IV) oxide nanopowder (21 nm) was obtained from Sigma-Aldrich. TiOSO_4_·*x*H_2_O + H_2_SO_4_ was purchased from Alfa Aesar (10 mesh, CAS: 13825-74-6). Trichloroethylene (TCE) was purchased from Sigma-Aldrich, and EPA standards were obtained from RESTEK (8260). All of the reagents were used as received. Milli-Q ultrapure water (18 MΩ cm) was used in all experiments, and all glassware was acid washed prior to experiments.

### Synthesis of TiO_2_ Particles

For flower-like particles, 25 mL of 0.5 M NaOH was added to a clear solution of 12.5 mL of 0.31 M TiOSO_4_·*x*H_2_O + H_2_SO_4_ (Figure 2, Step 2). For rod-like nanoparticles, 12.5 mL of 0.5 M NaOH was added to the same concentration of precursor solution. Following this step, all syntheses proceeded under identical conditions. The solution was stirred overnight, allowing a white sol to form and precipitate. After the mixture was centrifuged at 5000 rpm for 5 min, it was decanted and resuspended in ∼35 mL of water. This process was repeated 3 times. 6 M HNO_3_ and 6 M NaOH were used to adjust the pH of each mixture to the final desired pH: 1, 3, 6, 7, 10, and 12.5 (Figure 2, Step 4). The solutions were placed in a 50 mL PPL-lined hydrothermal autoclave and heated in a furnace/oven at 220 °C for 24, 48, 72, and 96 h. After being cooled, the pristine brookite TiO_2_ particles were filtered and washed with DI H_2_O.

### Characterizations

Powder XRD patterns were obtained by a Rigaku Miniflex II benchtop diffractometer over a 2θ range of 15–70° via Cu Kα radiation of 30 kV and 15 mA, and pattern collection was completed with a scan speed of 3° per min and a sampling width of 0.06°. Rietveld refinements on each pattern were undertaken by utilizing the PDXL2 analysis software. Anatase TiO_2_ PDF#00-064-0863 and brookite TiO_2_ PDF#01-076-1934 reference patterns were utilized in data analysis. Further testing was performed on Bruker D2 PHASER Benchtop XRD using a 2θ range of 15–70° via Cu Kα radiation of 30 kV and 10 mA, and the data were processed with DIFFRAC.EVA data analysis software with the Crystallography Open Database (rev. 278581). Morphologies of the final samples were analyzed by SEM, SEM-JSM-IT700HR, 15 kV, JEOL. Photocatalysis was performed in a Rayonet Reactor, and TCE analysis was accomplished using a Shimadzu GC/MS QP2010 Plus with a Restek Rtx-624 column and a Teledyne Tekmar Stratum Purge and Trap. The samples were run in accordance with the EPA standard, method 524.2. To generate the calibration curve, each sample run included 5 mL of ultrapure deionized water, 10 μL of a 15 ppm internal standard, 1,4-difluorobenzene, and varying concentrations of TCE standards ranging from 5 to 50 ppm. Subsequently, the areas under the peaks were quantified, and a calibration curve was constructed by plotting the ratio of TCE to the internal standard, TCE/IS, against the TCE concentration.

### Photocatalysis

Photocatalytic degradation of TCE was performed under 240 nm UV light (UVC range) in a Rayonet Reactor. The aqueous solutions were analyzed at desired illumination intervals (∼0, 60, and 120 s). Approximately 2 mg (±0.02 mg) of TiO_2_ nanoparticles was added to a 25 mL quartz vial with a known concentration of TCE, typically ranging from 25 to 45 ppm. Each vial was wrapped in foil and stored in the refrigerator overnight to equilibrate. The next day, the solution was sonicated and placed into a photoreactor. Five mL of the solution at a time was analyzed by GC/MS with 10 μL of 15 ppm of IS. Afterward, the areas under the peaks for both the IS and TCE were quantified. Using the calibration curve equation, the TCE concentrations were calculated. To construct the photodegradation curves, the TCE concentrations were normalized as C_i_/C_0_, where C_0_ is the initial concentration and C_i_ is the concentration at each time point during degradation by nanoparticles. Triplicate runs were performed for all samples to obtain standard deviation values.

## Supplementary Material



## Notes

XRD patterns of purchased TiO_2_ nanoparticles. XRD patterns of TiO_2_ nanoparticles formed at pH 10.5. pH 1. and pH 6 and pH 7 .heated at different temperatures. XRD patterns of samples prepared with KOH. SEM images of TiO_2_ nanoparticles synthesized at different temperatures and with different NaOH concentrations, average peak position of the doublet brookite diffraction peaks and their corresponding crystallite sizes, SEM images of purchased TiO_2_ nanoparticles, and photodegradation of TCE with purchased TiO_2_ nanoparticles (PDF)
