# Supplementary material for: Insights into Successful Hydrothermal Synthesis of Brookite TiO2 Particles: From Micro to Nano
Source: ACS Omega. 2025 Nov 4;10(45):54160–6. doi: 10.1021/acsomega.5c06112 (PMC12631344; doi:10.1021/acsomega.5c06112)
Supplement: Supplementary file 1 [file ao5c06112_si_001.pdf]

Supporting Information

Title: Insights Into Successful Hydrothermal Synthesis of Brookite TiO_2 Particles: From Micro to Nano.

Authors: Luke T. Coward; Nataliya Stynka; Victoria G. Magyar; Ava Foreman; Luca Antonescu; Hanna McFadden; Lorelei Dippy; Jocelyn D. Shutak; Madeline Kesner; Shawn Overcash; Joshua Davis; Camila Rendon Bernot; Oksana Love.*

University of North Carolina Asheville; One University Heights, United States, Asheville NC 28804

*Corresponding Author: olove@unca.edu

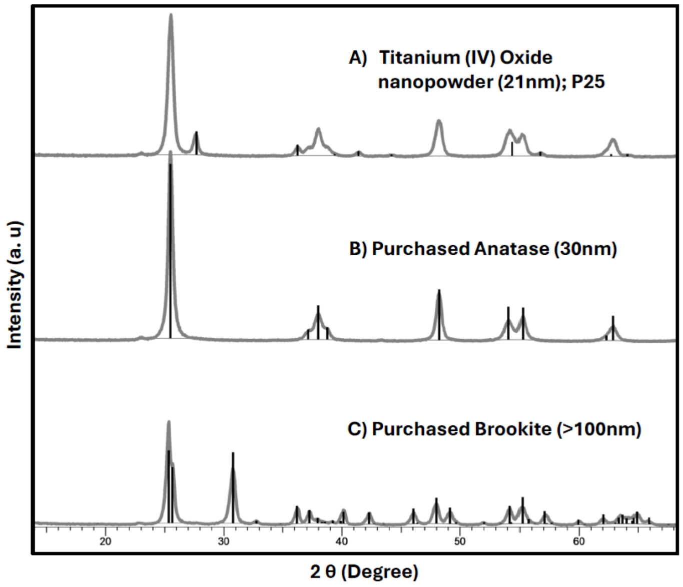

Figure S1. XRD patterns of A) Titanium (IV) Oxide (21nm) nanopowder (COD 9004143 Rutile), B) purchased anatase nanoparticles 30nm (COD 9009086 Anatase), and C) purchased brookite nanoparticles (COD 9004138 Brookite).

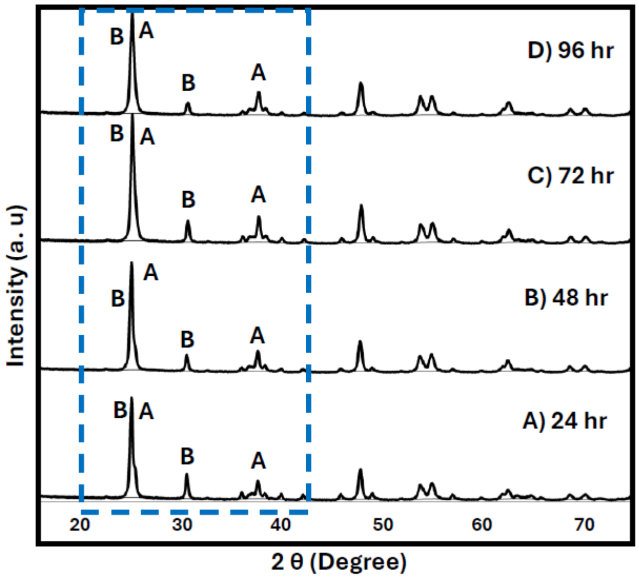

Figure S2. XRD patterns of the samples prepared at final pH 10.5 heated at A) 24 hours, B) 48 hours, C) 72 hours, and D) 96 hours. Mixture of anatase and brookite was present in the samples, while more anatase character was observed with longer heating times.

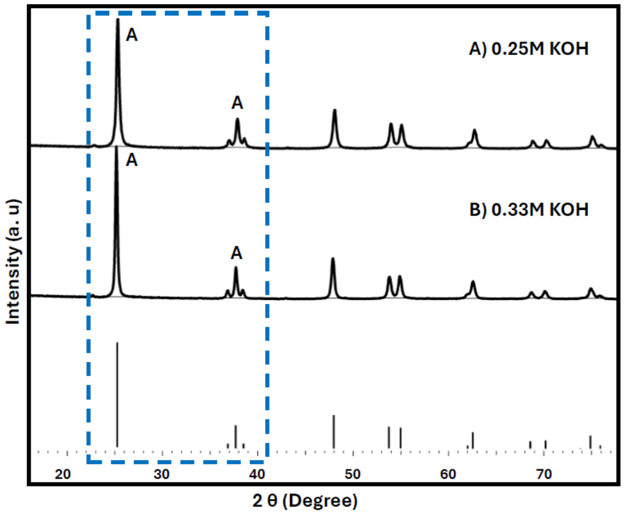

Figure S3. XRD patterns of the samples prepared with A) 0.25M KOH, and B) 0.33M KOH. COD 5000223 for Anatase. For A) 25mL of 0.5M KOH was added to a clear solution of 12.5 mL of 0.31M $TiOSO_4{\cdot}xH_2O$ +H_2SO_4 (Figure 2, Step 2). For B)12.5mL of 0.5M KOH was added to the same concentration of precursor solution. Following this step, all syntheses proceeded under identical conditions as described in Experimental Section.

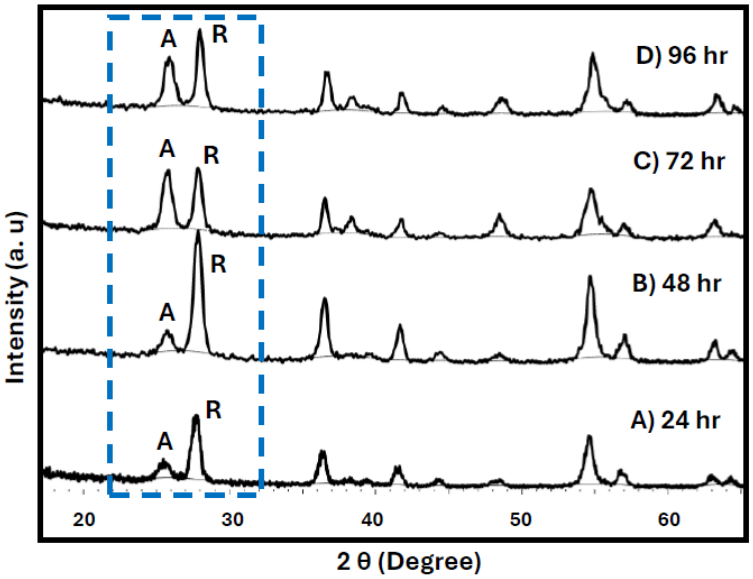

Figure S4. XRD patterns of the samples prepared at final pH 1 heated at A) 24 hours, B) 48 hours, C) 72 hours, and D) 96 hours. Mixture of anatase and rutile was present in the samples, while more anatase character was observed with longer heating times.

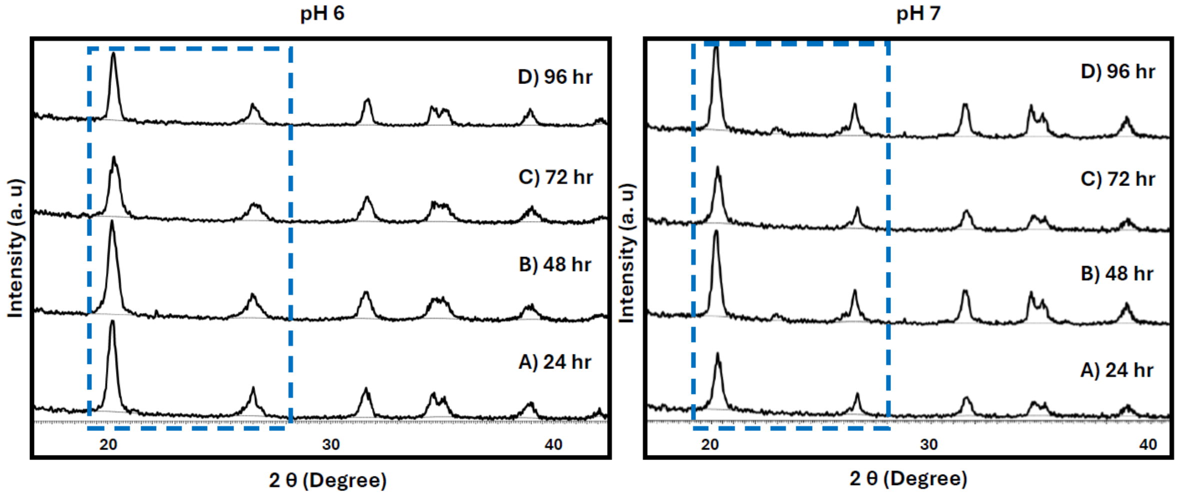

Figure S5. XRD patterns of the samples prepared at final pH 6 (left) and pH 7 (right) heated at A) 24 hours, B) 48 hours, C) 72 hours, and D) 96 hours. Pure anatase phase was created for all samples.

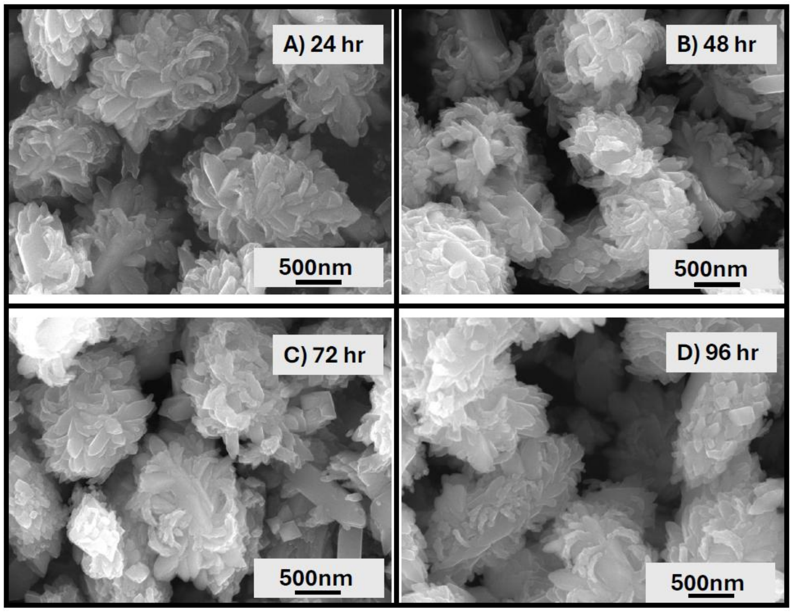

Figure S6. SEM images of brookite TiO_2 particles at A) 24 hours, B) 48 hours, C) 72 hours, and D) 96 hours of heating at 220°C.

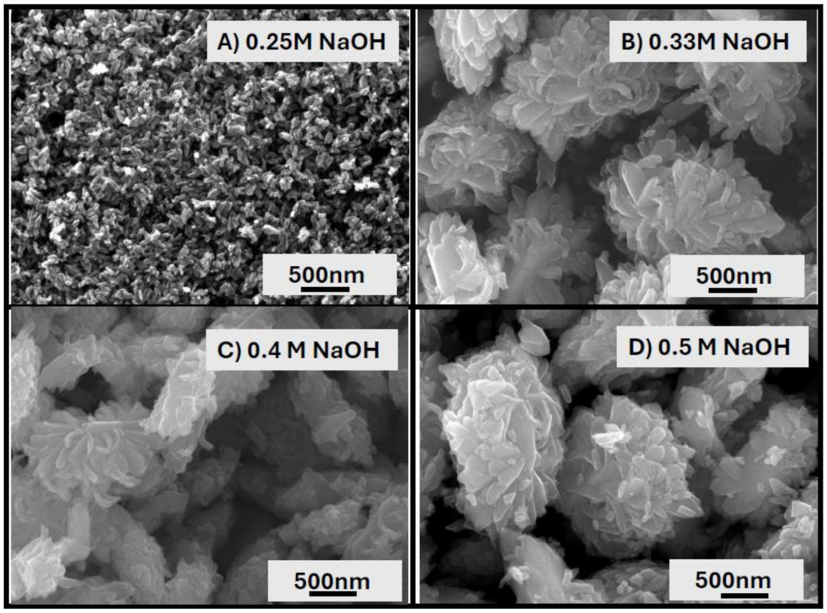

Figure S7. SEM images at 500nm scale of the samples prepared with 12.5 mL of 0.31M $TiOSO_4{\cdot}xH_2O$ +H_2SO_4 and A) final 0.25 M NaOH obtained from addition of 0.5M 12.5 mL NaOH, B) final 0.33 M NaOH obtained from addition of 0.5M 25 mL NaOH C) final 0.4 M NaOH obtained from addition of 0.5M 50 mL NaOH, and D) final 0.5 M NaOH obtained from addition of 1M 12.5 mL NaOH. All samples were heated for 24 hours at 220°C and followed previously stated purification techniques.

Average Peak Position of the Doublet Brookite Diffraction Peaks and Their Corresponding Crystallite Sizes

	1st Doublet Peak (2θ Degree)	Average Crystallite Size (nm)	2nd Doublet Peak (2θ Degree)	Average Crystallite Size (nm)
A) 0.25M NaOH	25.2	39.7	25.5	45.3
B) 0.33M NaOH	25.2	38	25.6	45.6
C) 0.4M NaOH	25.1	24.8	25.5	42.1
D) 0.5M NaOH	25.1	24.8	25.5	42.1

Table S1. The average peak position of the doublet brookite diffraction peaks and their corresponding crystallite sizes.

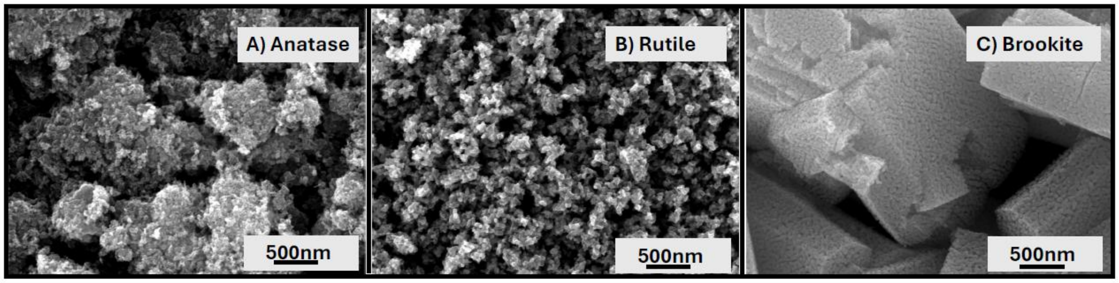

Figure S8. SEM images of purchased nanoparticles: A) Anatase (15nm), B) Rutile (30nm), and C) Brookite (>100nm).

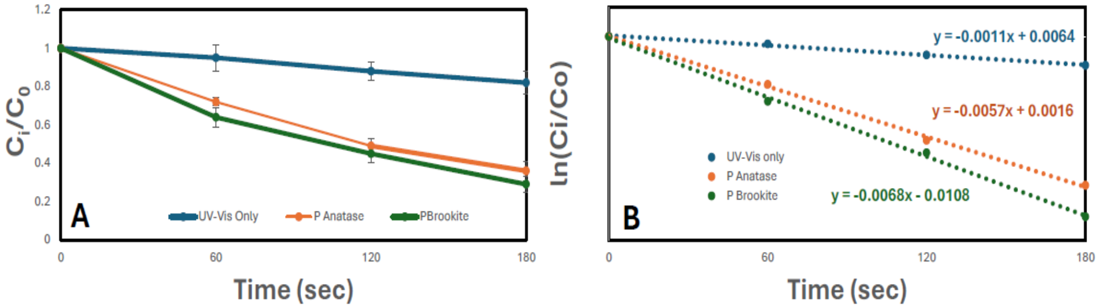

Figure S9. A) Degradation of TCE under UV light with purchased anatase and brookite TiO_2 nanoparticles; B) Reaction rates (1st order) of degradation of TCE by UV light, purchased anatase and brookite TiO_2 nanoparticles.
